# Diagnosis of Ovarian Neoplasms Using Nomogram in Combination With Ultrasound Image-Based Radiomics Signature and Clinical Factors

**DOI:** 10.3389/fgene.2021.753948

**Published:** 2021-09-28

**Authors:** Lisha Qi, Dandan Chen, Chunxiang Li, Jinghan Li, Jingyi Wang, Chao Zhang, Xiaofeng Li, Ge Qiao, Haixiao Wu, Xiaofang Zhang, Wenjuan Ma

**Affiliations:** ^1^ Department of Pathology, Tianjin Medical University Cancer Institute and Hospital, Tianjin, China; ^2^ National Clinical Research Center for Cancer, Tianjin, China; ^3^ Key Laboratory of Cancer Prevention and Therapy, Tianjin, China; ^4^ Tianjin’s Clinical Research Center for Cancer, Tianjin, China; ^5^ Department of Ultrasonographic Diagnosis and Therapy, Tianjin Medical University Cancer Institute and Hospital, Tianjin, China; ^6^ Department of Ultrasonographic Diagnosis and Therapy, Tianjin Ninghe Hospital, Tianjin, China; ^7^ Department of Bone and Soft Tissue Tumors, Tianjin Medical University Cancer Institute and Hospital, Tianjin, China; ^8^ Department of Molecular Imaging and Nuclear Medicine, Tianjin Medical University Cancer Institute and Hospital, Tianjin, China; ^9^ Department of Clinical Laboratory, Tianjin Medical University General Hospital, Tianjin, China; ^10^ Department of Breast Imaging, Tianjin Medical University Cancer Institute and Hospital, Tianjin, China

**Keywords:** radiomics, serous ovarian tumor, ultrasound, classification, nomogram, image analysis

## Abstract

**Objectives:** To establish and validate a nomogram integrating radiomics signatures from ultrasound and clinical factors to discriminate between benign, borderline, and malignant serous ovarian tumors.

**Materials and methods:** In this study, a total of 279 pathology-confirmed serous ovarian tumors collected from 265 patients between March 2013 and December 2016 were used. The training cohort was generated by randomly selecting 70% of each of the three types (benign, borderline, and malignant) of tumors, while the remaining 30% was included in the validation cohort. From the transabdominal ultrasound scanning of ovarian tumors, the radiomics features were extracted, and a score was calculated. The ability of radiomics to differentiate between the grades of ovarian tumors was tested by comparing benign vs borderline and malignant (task 1) and borderline vs malignant (task 2). These results were compared with the diagnostic performance and subjective assessment by junior and senior sonographers. Finally, a clinical-feature alone model and a combined clinical-radiomics (CCR) model were built using predictive nomograms for the two tasks. Receiver operating characteristic (ROC) analysis, calibration curve, and decision curve analysis (DCA) were performed to evaluate the model performance.

**Results:** The US-based radiomics models performed satisfactorily in both the tasks, showing especially higher accuracy in the second task by successfully discriminating borderline and malignant ovarian serous tumors compared to the evaluations by senior sonographers (AUC = 0.789 for seniors and 0.877 for radiomics models in task one; AUC = 0.612 for senior and 0.839 for radiomics model in task 2). We showed that the CCR model, comprising CA125 level, lesion location, ascites, and radiomics signatures, performed the best (AUC = 0.937, 95%CI 0.905–0.969 in task 1, AUC = 0.924, 95%CI 0.876–0.971 in task 2) in the training as well as in the validation cohorts (AUC = 0.914, 95%CI 0.851–0.976 in task 1, AUC = 0.890, 95%CI 0.794–0.987 in task 2). The calibration curve and DCA analysis of the CCR model more accurately predicted the classification of the tumors than the clinical features alone.

**Conclusion:** This study integrates novel radiomics signatures from ultrasound and clinical factors to create a nomogram to provide preoperative diagnostic information for differentiating between benign, borderline, and malignant ovarian serous tumors, thereby reducing unnecessary and risky biopsies and surgeries.

## Introduction

Histologically, serous tumors are the most prevalent ovarian tumors, representing 70% of the cases. (Javadi et al., 2016; Brett et al., 2017; Lheureux et al., 2019; Lisio et al., 2019). Such tumors can be classified into benign, borderline, and malignant lesions that exhibit distinct clinicopathological characteristics owing to which they exhibit differences in terms of therapeutic schemes, and prognoses. Benign tumors, which are usually slow-growing, respond well to conventional treatments. In contrast, the borderline serous ovarian tumors might be malignant potential, necessitating fertility-sparing surgery for fertile women who desire it. (du Bois et al., 2016; Chui et al., 2019). Moreover, therapy for ovarian cancer usually involves surgery and platinum/taxane doublet-based chemotherapy. (Lisio et al., 2019; Kuroki and Guntupalli, 2020). The diagnosis of serous ovarian tumors is difficult without incisional or aspiration biopsy. However, the varied characteristics of the serous ovarian tumors make it challenging to diagnose between borderline and malignant ovarian tumors using fine-needle aspiration. (Kuroki and Guntupalli, 2020). Therefore, it is crucial to develop a non-invasive and accurate preoperative identification technique for ovarian tumors for appropriate treatment planning by avoiding inadequate excision or surgical overtreatment, especially for premenopausal patients wanting to retain their fertility.

Adnexal ultrasound, a non-invasive, low-cost, and safe procedure, is currently the first-line imaging modality for ovarian tumor screening and diagnosis. Even though such pattern-recognition-based classification of ovarian masses into benign or malignant tumors demands much expertise, (Van Holsbeke et al., 2010; Dakhly et al., 2019)^,^ there is a shortage of expert examiners. Radiomics offers automatic extraction of mineable high-dimensional quantitative data from clinical images, thereby bypassing the need for human intervention, and shows great promise in tumor detection, diagnosis, and prognostic evaluation. (Chiappa et al., 2020; Mayerhoefer et al., 2020). Several researchers have recently employed radiomics features based on MRI, CT and ultrasound to evaluate the clinical outcomes of ovarian cancer patients. (Rizzo et al., 2018; Lu et al., 2019; Zhang et al., 2019; Veeraraghavan et al., 2020; Yao et al., 2021).

This study utilizes a two-step radiomics classification of serous ovarian tumors based on the imaging and builds a nomogram combining the clinical factors to distinguish benign, borderline, and malignant ovarian tumors.

## Materials and Methods

### Patients and Study Design

**FIGURE 1:**
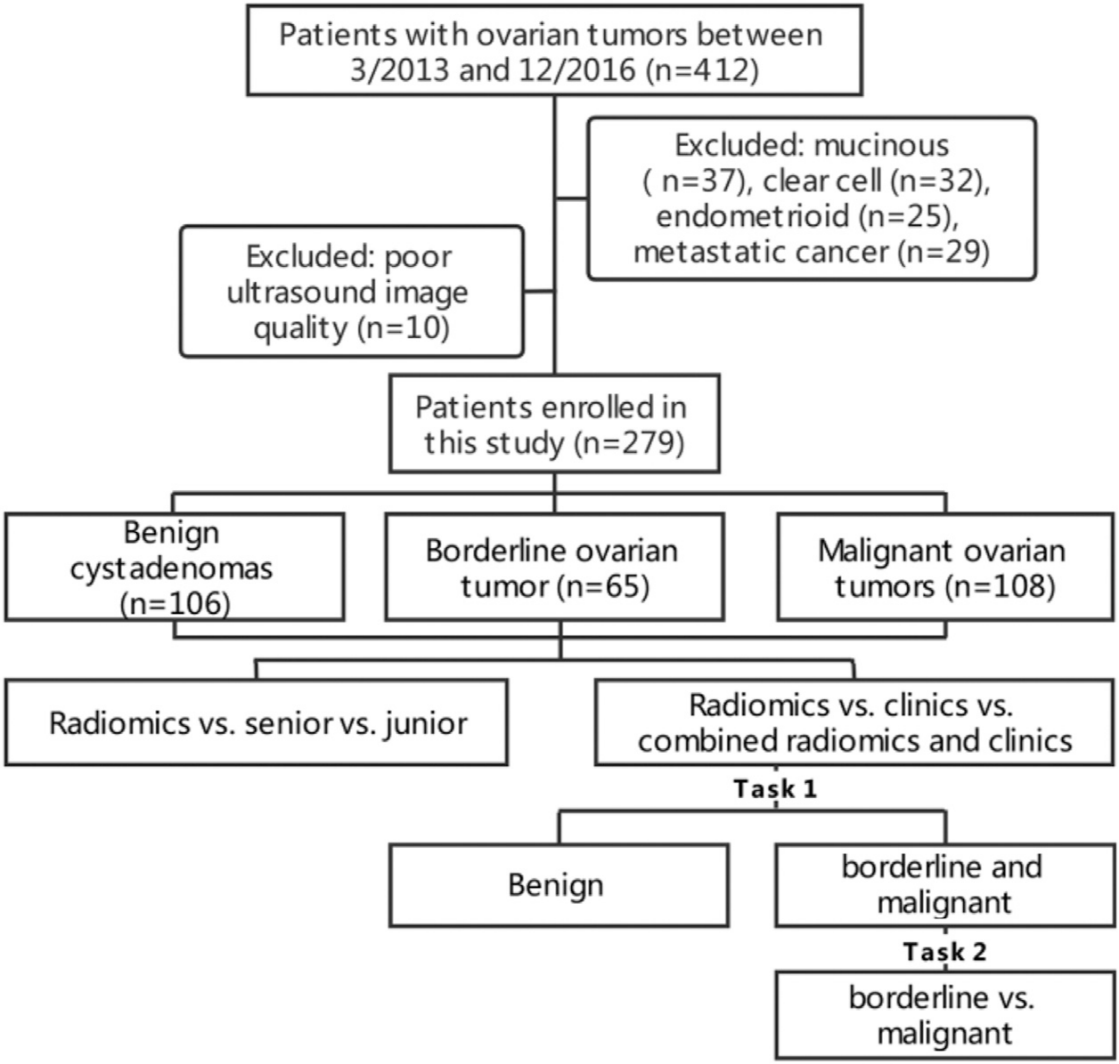
Flowchart of patient recruitment and experiments design.

This study was in accordance with the Declaration of Helsinki. The Ethics Committee of Tianjin Medical University Cancer Hospital approved this retrospective study (Approval No. bc2021114), and informed consent was waived. All the clinical and biodatas have been anonymized. We enrolled 412 patients with ovarian tumor from Tianjin Medical University Cancer Institute and Hospital (Tianjin, China). All patients were enrolled between March 2013 to December 2016. Patients with mucinous tumor, endometrioid tumor, clear cell cancer, metastatic cancer and the tumor with poor quality ultrasound images were excluded from the study. In total, 265 patients meeting the inclusion criteria were enrolled consecutively in our study. The samples comprised 106 ovarian cystadenomas, 65 borderline tumors, and 108 ovarian malignancies, all of which were pathologically confirmed to be serous. Of the tumors we eventually included, the ultrasound images of 28 tumors were from 14 patients who had bilateral ovarian tumor (7 patients with bilateral borderline serous tumors, seven patients with bilateral ovarian serous cancer). The patient data included age, age at menarche, CA125 level (range: 5.11–5000 IU/L), location of the lesion (unilateral or bilateral), family history of cancer, and ascites. The inclusion criteria were as follows: 1) histological diagnosis of benign, borderline, and malignant ovarian serous tumors; 2) availability of preoperative US images suitable for diagnostic analysis; 3) US scanning performed before neoadjuvant therapy or surgical resection. The exclusion criteria included the following: 1) no US results or the ovarian mass was not completely visible in the image; 2) mucinous, clear cell, endometrioid, metastatic cancer (Figure 1).

In a two-step decision-making approach, two tasks were performed to train and validate the ability to distinguish between benign vs borderline and malignant (task 1) and borderline vs malignant (task 2). A clinical-feature alone model and a combined clinical-radiomic (CCR) model were built using predictive nomograms for each task. During the development of these tasks, we used a fixed 70%/30% training/validation cohort split. A 10-fold cross-validation was done to evaluate the true diagnostic potential of this method.

### Ultrasound Imaging and Segmentation

**FIGURE 2:**
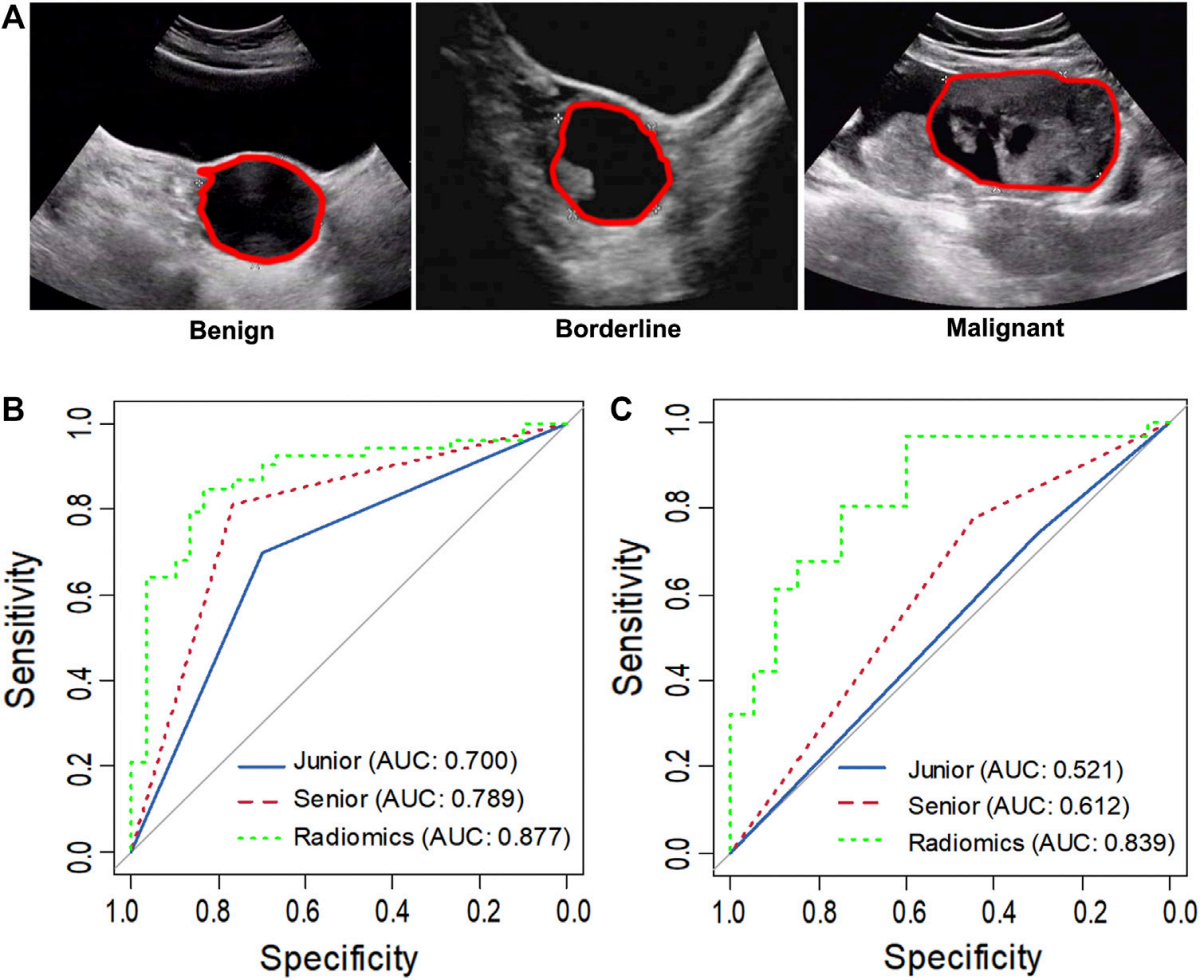
Representative ultrasound images of benign, borderline and malignant ovarian serous tumors **(A)**. The asterisk indicates the tumor boundary. The red marker line indicates the region of interest (ROI). ROC curve analysis comparing the diagnosis of the senior and junior sonographer and radiomics in task 1 **(B)** and task 2 **(C)**.

All ultrasound images were acquired using a Philips iU22/HD11 (California, United States) ultrasound machine with a 5–12 MHz probe and retrieved from the picture archiving and communication systems (PACS) for image segmentation and analysis at our institution (Figure 2A). The boundary of lesions manually segmented using ImageJ (https://imagej.nih.gov/ij/) by a sonographer with more than 8 years of experience. When the boundary was not determined, another experienced sonographer was consulted for a final opinion. The two sonographers were blind to the pathological and clinical information.

### Radiomics Signature Construction

Eight hundred and fifty-five radiomics features, including shape, gray-scale histograms, texture, and wavelet features, were extracted automatically from each segmented region of interest using an in-house software written in MATLAB R2018b (MathWorks, Inc., Natick, Massachusetts). Detailed information on the feature extraction algorithms is provided in Supplemental Table S1.

For each task, we followed a three-step procedure to identify the reliable radiomic features. First, the Wilson test was used to identify features highly related to the biomarkers with a significance of less than 0.05 (*p* < 0.05). Pearson correlation matrices were used to assess the correlation between the features where a correlation coefficient greater than 0.8 was considered redundant. One of two features with a lower *p*-value was excluded. Next, the minor absolute shrinkage and selection operator (LASSO) regression method was used to select the most useful prognostic combination of features followed by the computation of the radiomics score (Radscore) for each patient through a linear combination of selected features weighted by their respective coefficients.

### Human Readout

All images from the validation cohort were in random order subjected to critical evaluation by a senior (LCX, with 8 years of working experience) and a junior sonographer (LJH, with 2 years of working experience) in the ultrasound department, where each of them had carried out over 200 scans of ovarian ultrasound images per year. Both readers were blinded to the clinical information, study design, and background.

### Nomogram Construction

Clinical factors, including age, CA125 level, lesion location, family history of cancer, ascites, and Radscore, were evaluated using univariate analysis in the training set. Variables with *p* < 0.05 in the univariate analysis were included in the multivariate logistic analysis. The clinical and CCR models were built using these clinical variables with or without a Radscore for each task. These models were presented in the form of a nomogram.

### Statistical Methods

In this study, the continuous variables were presented as the mean (± standard deviation), and categorical variables were recorded as numbers and percentages. The chi-square test, Fisher’s exact test, or Wilcoxon sum-rank test were used to identify categorical variables for the univariate analysis. Binary logistic regression analysis was used for multivariate analysis. Based on the factors mentioned above, the multivariate logistic regression model was adopted to establish two nomograms for diagnosing ovarian neoplasms: clinical-feature alone model vs CCR model. The performance of the nomogram was evaluated based on diagnostic accuracy, sensitivity, and specificity of receiver operating characteristic (ROC) curves and calibration curves. The difference in the area under the curve (AUC) between the training and validation datasets was tested using the *p*-value of integrated discrimination improvement (IDI) and Delong’s (D) test, and the 95% confidence intervals (CI) were calculated.

All statistical analyses were conducted using the R software (version 6.1, R Foundation for Statistical Computing, Vienna, Austria). A two-tailed difference was considered significant at *p* < 0.05.

## Results

### Evaluation of the Clinical Parameters of the Patients

The clinical features of patients in the training and validation cohorts for the two tasks were summarized in Tables 1, 2. We observed a significant difference in the CA125 level, lesion location, and ascites between benign and non-benign serous ovarian lesions in the training cohort (Table 1). As shown in Table 2, age, CA125 level, and ascites significantly differed between the borderline and malignant serous ovarian tumors.

**TABLE 1:** Clinical characteristics of patients in training and validation cohorts in task 1

Characteristics	Training cohorts		*p*-value			Validation cohorts		*p-*value
	Benign (n = 76)	Non-benign (n = 120)	Univariate analysis		Multivariate analysis	Benign (n = 30)	Non-benign (n = 53)
**Age** ^#^	51.2 ± 13.4	48.0 ± 13.5	0.102		—	49.1 ± 16.1	49.7 ± 11.2	0.861
**Age at menarche** ^#^	14.6 ± 1.77	14.6 ± 1.85	0.869		—	14.6 ± 1.52	14.7 ± 1.69	0.119
**CA125 level (IU/L), No (%)**	—	—	<0.001*		<0.001*	—	—	<0.001*
0	75 (98.7)	73 (0.6)	—		—	0 (0.0)	26 (49.1)	—
1	1 (1.3)	47 (0.4)	—	—		30 (100.0)	27 (50.9)	—
**Tumor side, No (%)**		—	<0.001*	0.002*		—	—	<0.001*
Bilateral	15 (19.7)	69 (56.7)	—	—		4 (13.3)	34 (64.5)	—
Unilateral	61 (80.3)	52 (43.3)	—	—		26 (86.7)	19 (35.9)	—
**Family history of cancer, No (%)**		—	0.161	—		—	—	0.789
Yes	14 (18.4)	34 (28.3)	—	—		8 (26.7)	17 (32.1)	—
No	62 (81.7)	86 (71.7)	—	—		22 (73.3)	36 (67.9)	—
**Ascites, No (%)**	—	—	<0.001*	<0.001*		—	—	0.001*
Yes	0 (0.0)	39 (32.5)	—	—		30 (100.0)	36 (67.9)	—
No	76 (100.0)	81 (67.5)	—	—		0 (0.0)	17 (32.1)	—

Note: Non-benign, borderline and malignant tumors, # mean ± SD, ≤500 IU/L, 0; >500 IU/L, 1. SD, standard deviation. *p value < 0.05.

**TABLE 2:** Clinical characteristics of patients in training and validation cohorts in task 2

Characteristics	Training cohorts		*p-*value		Validation cohorts		*p-*value
	Borderline (n = 45)	Malignant (n = 77)	Univariate analysis	Multivariate analysis	Borderline (n = 20)	Malignant (n = 31)

**Age** ^#^	43.8 ± 14.0	52.3 ± 9.06	<0.001*	<0.001*	36.7 ± 13.0	53.7 ± 12.2	<0.001*
**Age at menarche** ^#^	14.2 ± 2.02	14.8 ± 1.49	0.084	—	14.0 ± 1.86	15.2 ± 1.95	0.039*
**CA125 level (IU/L), No (%)**		—	0.001*	0.003*	—	—	0.312
0	35 (77.8)	35 (45.4)	—	—	6 (30.0)	15 (48.4)	—
1	10 (22.2)	42 (54.5)	—	—	14 (70.0)	16 (51.6)	—
**Tumor side, No (%)**		—	0.432	—	—	—	1
Bilateral	24 (53.3)	48 (62.3)	—	—	12 (60.0)	18 (58.1)	—
Unilateral	21 (46.7)	29 (37.7)	—	—	8 (40.0)	13 (41.9)	—
**Family history of cancer, No (%)**		—	0.639	—	—	—	0.201
Yes	12 (26.7)	25 (32.5)	—	—	3 (15.0)	11 (35.5)	—
No	33 (73.3)	52 (67.5)	—	—	17 (85.0)	20 (64.5)	—
**Ascites, No (%)**	—	—	<0.001*	0.006*	—	—	0.125
Yes	5 (11.1)	33 (42.9)	—	—	16 (80.0)	17 (54.8)	—
No	40 (88.9)	44 (57.1)	—	—	4 (20.0)	14 (45.2)	—

Note: # mean ± SD. ≤500 IU/L, 0; >500 IU/L, 1. SD, standard deviation. *p value < 0.05.

### A Comparative Analysis of the Diagnostic Performances of the Radiomics Model, the Senior and Junior Sonographer

In task 1, LASSO was used to evaluate the diagnostic capability of 17 potential informative predictors (Supplementary Figures S1A, C), and the outputs were to Radscore calculation formula (Supplemental Material). We observed that the differences in the Radscore values between the benign and non-benign serous ovarian tumors in the training and validation cohorts were statistically significant (*p* < 0.001, Supplementary Figures S2A, B). The ROC curve analysis of the radiomics model showed AUCs of 0.907 (95% CI 0.863–0.950) and 0.877 (95% CI, 0.798–0.957) in the training and validation sets, respectively revealed no significant differences (D = 0.633; *p* = 0.5278). Next, we evaluated the diagnostic capability of the two sonographers to draw our comparative analysis. Figure 2B; Table 3; Supplementary Table S2 showed the diagnostic performance of the junior sonographer, senior sonographer, and radiomics model, respectively. A statistically significant difference between the junior sonographer and the radiomics model (D = 3.611; *p* < 0.001) was observed. However, there was no statistically significant difference between the performances of the senior sonographer and the radiomics model (D = 1.473; *p* = 0.141).

**TABLE 3:** Diagnostic performance comparison among the senior sonologist, the junior sonologist, radiomics, clinics and combination of radiomics and clinics in the validation cohort of each task.

		AUC (95%CI)	ACC (95%CI)	SEN (95%CI)	SPE (95%CI)
**Task 1**	senior	0.789 (0.695–0.883)	0.795 (0.692–0.876)	0.697 (0.511–0.838)	0.860 (0.726–0.937)
	junior	0.699 (0.595–0.803)	0.699 (0.588–0.795)	0.568 (0.396–0.725)	0.804 (0.656–0.901)
	Radiomics	0.877 (0.798–0.957)	0.843 (0.747–0.914)	0.758 (0.574–0.883)	0.900 (0.774–0.963)
	Clinics	0.855 (0.786–0.924)	0.807 (0.706–0.886)	0.684 (0.512–0.820)	0.911 (0.779–0.971)
	Combination	0.914 (0.851–0.976)	0.880 (0.790–0.941)	0.813 (0.630–0.821)	0.922 (0.803–0.975)
**Task 2**	senior	0.612 (0.478–0.747)	0.647 (0.501–0.776)	0.563 (0.306–0.792)	0.686 (0.506–0.826)
	junior	0.521 (0.392–0.650)	0.569 (0.423–0.707)	0.429 (0.188–0.703)	0.622 (0.448–0.771)
	Radiomics	0.839 (0.725–0.952)	0.824 (0.691–0.916)	0.923 (0.621–0.996)	0.790 (0.622–0.899)
	Clinics	0.829 (0.706–0.950)	0.784 (0.647–0.887)	0.714 (0.477–0.878)	0.833 (0.645–0.937)
	Combination	0.890 (0.794–0.987)	0.863 (0.737–0.943)	0.842 (0.595–0.958)	0.875 (0.701–0.959)

AUC area under the receiver operator characteristic curves, ACC accuracy, SEN sensitivity, SPE specificity.

In Task 2, 22 potential informative predictors were explored using the LASSO method (Supplementary Figures S1B, D). Differences in the Radscore value between the borderline and malignant serous ovarian tumors in the training and validation cohorts were statistically significant (*p* < 0.001, Supplementary Figures S2C, D). The ROC curves of the radiomics model showed AUCs of 0.891 (95% CI 0.833–0.950) and 0.839 (95% CI 0.725–0.952) in the training and validation cohorts, respectively, with no significant difference between them (D = 0.607; *p* = 0.546). Figure 2C; Table 3; Supplementary Table S2 showed the diagnostic performance of the junior sonographer, senior sonographer, and radiomics model, respectively. There was a statistically significant difference between the performances of the junior/senior sonographer and the radiomics model (senior: D = 3.5, *p* < 0.001; junior: D = -4.640, *p* < 0.001).

By comparing the results from the 10-fold cross-validation run of the models built above to the results obtained on the fixed training/validation split, we found that the performance estimates were comparable for both the tasks with no indication of substantial overfitting (Supplementary Table S3).

### Construction and Validation of the Nomogram

**FIGURE 3:**
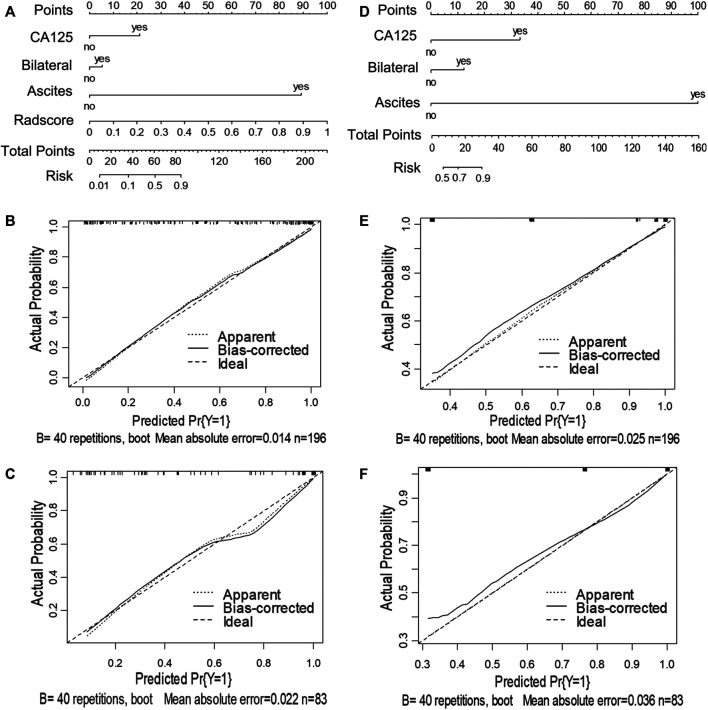
Nomograms and calibration curves of the combined clinical-radiomic (CCR) and clinical alone model in task 1. The nomograms were constructed in the training cohort. **(A)** Nomogram in the CCR model. **(B, C)** Calibration curves in the training and validation cohort of the CCR nomogram, respectively. **(D)** Nomogram in the clinical alone model. **(E, F)** Calibration curves in the training and validation cohort of the clinical alone nomogram, respectively.

**FIGURE 4:**
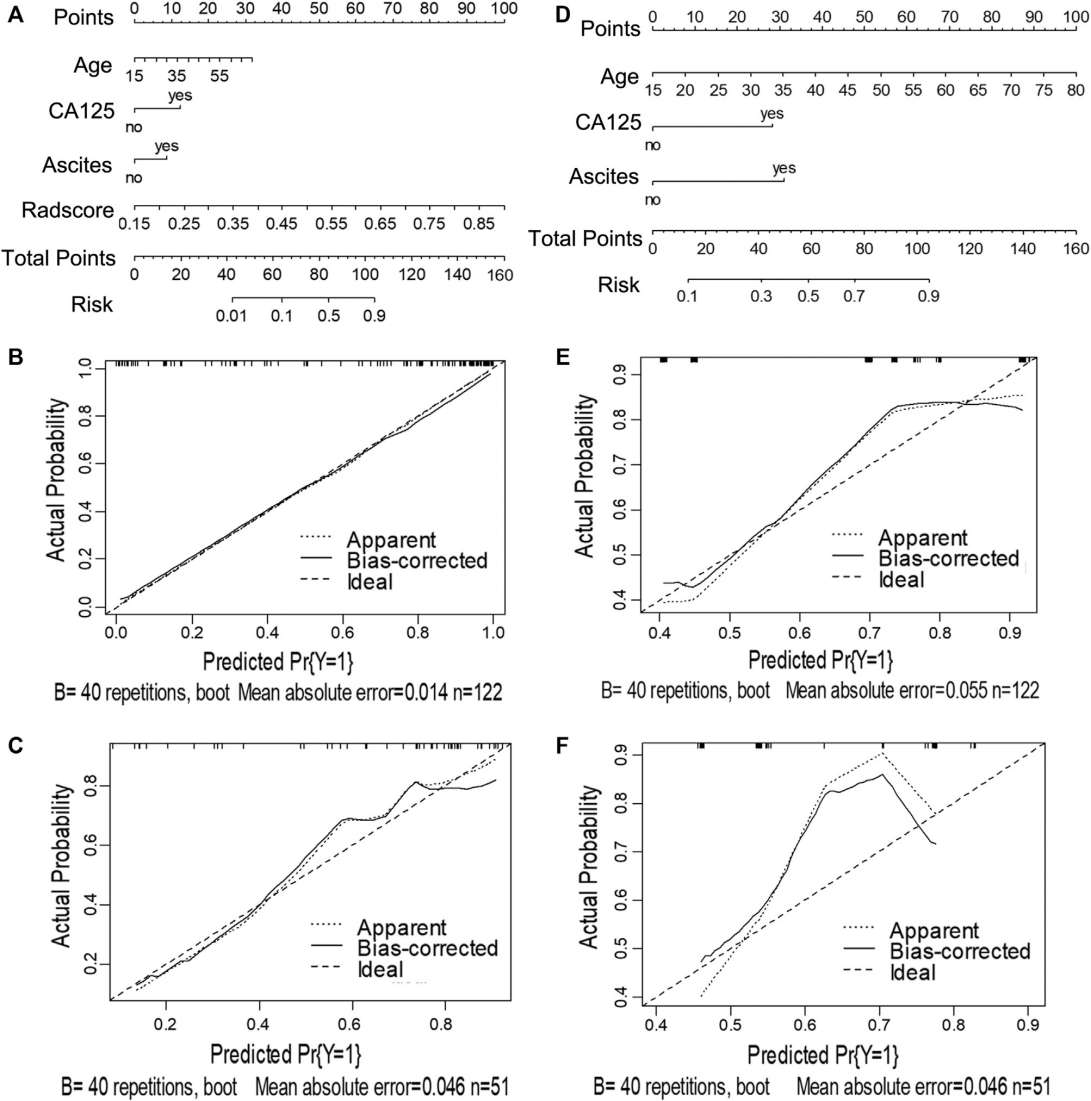
Nomograms and calibration curves of the CCR and clinical alone model in task 2. The nomograms were constructed in the training cohort. **(A)** Nomogram in the CCR model. **(B, C)** Calibration curves in the training and validation sets of the CCR nomogram, respectively. **(D)** Nomogram in the clinical alone model. **(E, F)** Calibration curves in the training and validation cohort of the clinical alone nomogram, respectively.

Next, we utilized the features mentioned above for each task to perform multivariate logistic regression analysis to construct the two models for diagnosing ovarian neoplasms, thereby leading to the generation of two nomograms, the clinical-feature alone model (Figure 3D and Figure 4D) and the combined clinical-radiomic (CCR) model (Figure 3A and Figure 4A).

**FIGURE 5:**
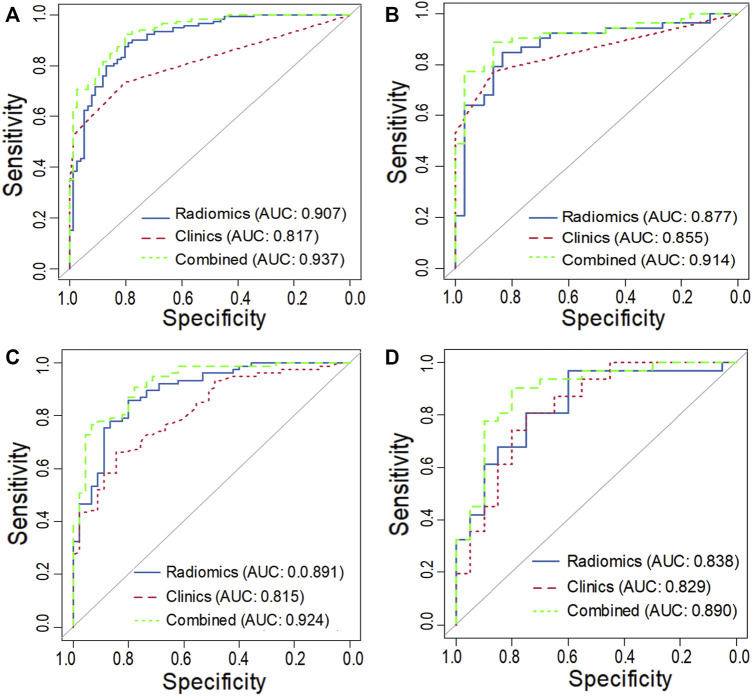
ROC curves of the training and validation cohort in task 1 **(A, C)** and task 2 **(B, D)**.

For task 1, Figure 3, Figure 5 and Table 3 showed the calibration curve and performance of the clinical-alone and CCR models. The ROC curves of the clinical-alone model showed AUCs of 0.817 (95% CI 0.765–0.868) and 0.855 (95% CI 0.786–0.924) in the training and validation cohorts, respectively (Figures 5A,C), with no significant difference between them (D = -0.88079; *p* = 0.3796). The ROC curves of the CCR model showed AUCs of 0.937 (95% CI 0.905–0.969) and 0.914 (95% CI 0.851–0.976) in the training and validation cohorts, respectively (Figures 5A,C), with no significant difference between them (D = 0.6394; *p* = 0.524). The calibration curve indicating the prediction from the two models (solid line) closely followed the 45-degree line in the training and validation cohorts, suggesting good diagnostic accuracy (Figures 3B,C for the CCR model and Figures 3E,F for the clinical alone model).

For task 2, the CCR performed satisfactorily in the training (AUC 0.924 [95% CI 0.876–0.971]) and the validation (AUC 0.890 [95% CI 0.794–0.987]) cohorts, respectively (Figures 5B,D), with no significant difference between them (D = 0.607; *p* = 0.546). The ROC curves of the clinical-feature alone model showed AUCs of 0.815 (95% CI 0.740–0.890) and 0.829 (95% CI 0.706–0.950) in the training and validation cohorts, respectively, with no significant difference between them (D = −0.189, *p* = 0.85). The calibration curve suggested good diagnostic accuracy for the CCR model (Figures 4B,C), which was slightly worse for the clinical-feature alone model (Figures 4E,F).

### Difference in the Prediction Performance Between the Clinical Alone Model and Combined Clinical-Radiomic Model

**FIGURE 6:**
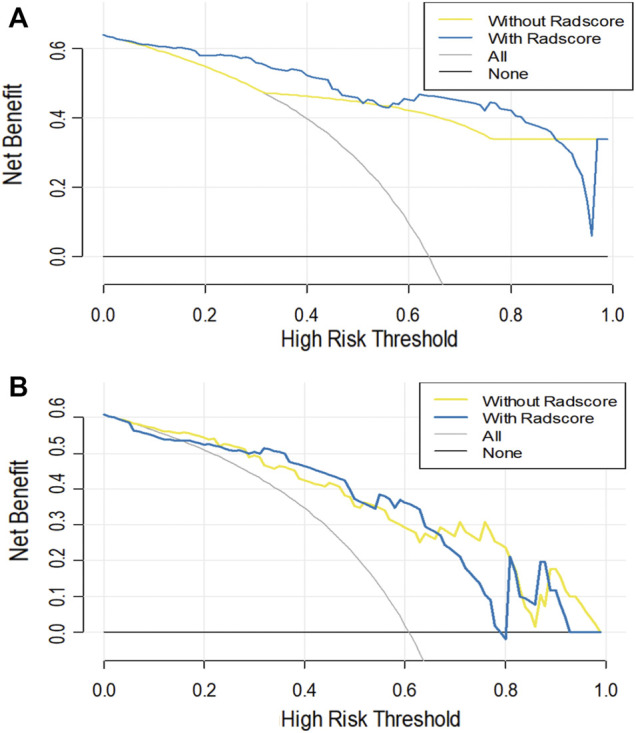
The decision curve analysis for the clinical alone model and the CCR model in task 1 **(A)** and task 2 **(B)**. The *Y*-axis represents the net benefit. The red line represents the clinical alone monogram. The green line represents the CCR model. The grey line represents the assumption that all patients have ovarian tumors. The black line represents the assumption that no patient has ovarian tumors.

As shown in Table 3 and DCA curves (Figure 6), the CCR model showed a relatively better predictive performance than the clinical-feature alone model for two tasks (task 1: IDI = 0.154, 95% CI: 0.078–0.231, *p* < 0.001; task 2: IDI = 0.815, 95% CI: 0.066–0.303, *p* = 0.002). The decision curves indicated that using the clinical features combined radiomics nomogram to predict types of serous ovarian cancer adds more benefit than the clinical-feature alone model.

## Discussion

In this study, we divided the three-class classified (benign vs borderline vs malignant tumors) ovarian neoplasms into two categories, i.e., benign vs borderline and malignant (task 1) and borderline vs malignant (task 2). First, two US-imaging-based radiomics models were established for each task. The diagnostic efficiency of the radiomics models was compared with that of junior and senior sonographers to evaluate their integrity. Both tasks of radiomics analysis showed satisfactory performance, especially in task 2, indicating higher accuracy than the experienced sonographer at identifying borderline ovarian tumors. Then, the combined clinical-radiomics CCR model was established for each task, where the CCR models significantly outperformed the clinical models.

To date, US-based examinations were considered the primary imaging technique for preoperative prediction of ovarian tumors. (Di Legge et al., 2017). Benign serous ovarian tumors are typically simple smooth-walled unilocular or multilocular cystic masses, (Virgilio et al., 2019), whereas serous borderline ovarian tumors tend to form cystic masses with profuse papillary projections. (Timor-Tritsch et al., 2019). Moreover, serous ovarian tumors form large, complex, solid, and cystic masses irregular, thick cystic walls with septations, necrosis, and solid mural nodules. (Moro et al., 2017). However, these imaging features are not specific and, to a certain extent are subject to the diagnostic experience of the sonographer. Nevertheless, conventional imaging evaluation by manual assessment of lesions by expert sonographers relying on semantic features provides a wealth of information on tumor heterogeneity, despite having a few drawbacks.

In this era of personalized and targeted oncology, radiomics enabled digitally encrypted medical images to be transformed into numerous quantitative features that provide information on tumor pathophysiology. (Bolton et al., 2012; Jiang et al., 2018; Mayerhoefer et al., 2020; Jian et al., 2021). To date, only one study has reported discriminating between benign and malignant ovarian tumors by computerized ultrasound image analysis using deep neural networks (DNNs). (Christiansen et al., 2021). However, distinguishing the borderline tumors using DNNs remains largely unexplored. Additionally, some reports have indicated that the MRI radiomics model can achieve higher accuracy in discriminating benign ovarian lesions from malignancies and between type I and type II ovarian epithelial cancer. (Zhang et al., 2019; Qian et al., 2020). Pan et al. developed a nomogram model that combined CT radiomics and semantic features, which could be used for imaging biomarkers (radiomic and semantic features) to classify serous and mucinous types of ovarian cystadenomas. (Pan et al., 2020). Song and colleagues established classification predictive tasks constructed from radiomics features extracted from dynamic contrast-enhanced magnetic resonance imaging (DCE-MRI) pharmacokinetic protocol from 104 ovarian lesions to discriminate between benign, borderline, and malignant ovarian tumors. In consistence with our results, radiomics analysis based on the DCE-MRI pharmacokinetic protocol demonstrated good differentiation between benign, borderline and malignant ovarian tumors in both two- and 3-class classification predictive tasks. (Song et al., 2020). To our knowledge, this is the first attempt to predict benign, borderline, and malignant ovarian serous tumors using radiomics features based on US images. The results of the 10-fold cross-validation confirmed those performance estimates, indicating no substantial overfitting.

Imaging features alone are often insufficient to determine the diagnosis and management of ovarian neoplasms. Hence, clinicians also consider the clinical context, including age, serological indicators, and familial risk factors, to make decisions. CA125 could serve as a critical serum biomarker for diagnosing and monitoring the relapse of serous ovarian cancer. (Matulonis et al., 2016). Ascites contain various cellular and acellular components that are known to facilitate metastasis and contribute to chemoresistance in ovarian serous cancer. (Ford et al., 2020). It is known that age is one of the most important poor prognostic markers for ovarian cancer. The incidence of ovarian cancer in women under 55 years of age is lower than that in women older than 55 years (Ma et al., 2019). Borderline and malignant serous ovarian tumors are more likely to occur in both ovaries. As expected, in this study cohort, the CA125 level was higher in the borderline and malignant serous ovarian tumor group than in the benign group. More borderline and malignant serous ovarian tumor cases were associated with ascites and showed involvement of both ovaries. (Jayson et al., 2014; Gershenson, 2017). We included these easily obtained clinical risk factors and US-based radiologic factors together with CA125 levels in our model development process. The improved nomogram model performed significantly better than the radiomics model or clinical model alone. The success of the nomogram model supported the idea that combining imaging features with complementary information from clinical reports that reflect the global outlook of the tumor is more helpful in the differential diagnosis of benign, borderline, and malignant serous ovarian tumors.

It is worth noting that the associations between the clinical variables and pathological diagnosis were discrepant in the training and validation cohorts. For example, CA125 level and ascites showed *p* values less than 0.05 in the training cohort, but they were not significantly associated with pathological diagnosis in the validation cohort of task 2. This result shows that clinical factors may be vulnerable to variations in data sets. However, radiomics features were consistently associated with pathological diagnosis and had accurate discriminative ability across all datasets.

However, the present study has some limitations. First, this was a retrospective study conducted in a single hospital with limited sample size. External multi-center validation in a larger cohort is needed in the future to improve the radiomics analysis. Second, because ovarian tumors comprise benign, borderline, and malignant lesions, discrimination results among the three categories need to be obtained directly. Therefore, the need of the hour is a 3-class classification task of radiomics analysis based on US imaging, which will be developed in the future.

## Conclusions

In conclusion, the current study presents a nomogram constructed from the US-based radiomics signature, clinical risk factors, and serum biomarkers. It could provide complementary diagnostic information to differentiate between benign, borderline, and malignant ovarian serous tumors, thereby contributing to reducing the number of unnecessary and risky biopsies and surgeries.

## Data Availability

The original contributions presented in the study are included in the article/Supplementary files, further inquiries can be directed to the corresponding authors.
