# Supplementary material for: Diagnosis of Ovarian Neoplasms Using Nomogram in Combination With Ultrasound Image-Based Radiomics Signature and Clinical Factors
Source: Front Genet. 2021 Sep 28;12:753948. doi: 10.3389/fgene.2021.753948 (PMC8505695; doi:10.3389/fgene.2021.753948)
Supplement: Supplementary file 1 [file Presentation1.pdf]

Supplementary Material

1. US image feature extraction

To normalize the different image specifications from various US scanners, image resampling and gray-level normalization were performed before quantitative feature extraction. All image data were resampled at a 1×1×1-mm voxel space size. The quantitative features were extracted from ROIs using an in-house software developed with MATLAB 2018B (MathWorksInc.).

A total of 885 radiomic features were drawn from each segmented lesion and can grouped as follows: (1) Morphologic features: four metrics, including area, largest diameter, length to width ratio and roundness, were calculated for the morphological description of the images. Area is the number of voxels in the tumor region extracted from US images multiplied by the dimension of voxels. Largest diameter is the voxels number of the long axis. Length to width ratio is the ratio of length to width. Roundness is defined as the ratio of the circumcircle radius to the inscribed circle radius of the lesion ROI. (2) Gray-scale histograms features: three features were computed for each lesion according to the definitions of the gray-scale histogram: variance, skewness and kurtosis. Their definition can be found in literatures Rodenacker K (2003). (3) Texture features: in total, 40 texture features were extracted from the tumor regions of US images after wavelet transform. Table 1 presents the list of texture features used in this study. Detailed description and methodology employed to extract the texture features is available in M Valli ères (2015). (4) Wavelet features: wavelet transform effectively decouples textural information by decomposing the original image. In this study a discrete, one-level and undecimated two dimensional wavelet transform was applied to each US image, which decomposes the original image into 4 decompositions (LL, HL, LH and HH). For each decomposition we computed gray-scale histograms and the textural features as described in Table S1.

2. Radiomics score

Task 1

Rad-score was calculated by summing the selected features weighted by their coefficients. The final formula of rad-score is:

*Radscore=0.633696452+0.081990230*text_Ng_8_vox_1_glszm_GLV--0.513829758*text_Ng_8_vox_1_glszm_ZSV-0.184691241*text_Ng_32_vox_1_glszm_SZHGE+0.429918997*text_Ng_64_vox_1_glszm_GLV-0.077451561*wave_glszm_8_LL_SZE+0.198574585*wave_glrlm_8_HL_GLV-0.296143717*wave_glszm_8_HL_SZE-0.001275886*wave_glszm_8_LH_ZSN-*

*0.324045370*wave_glrlm_16_LL_SRLGE+0.062247981*wave_glszm_16_HL_GLN-0.415170635*wave_glszm_16_HL_ZSN-0.264894683*wave_glszm_32_LH_ZSV-0.027362814*wave_glszm_32_HH_ZSN-0.162106511*wave_ngtdm_32_HH_Complexity+0.090016740*wave_glrlm_64_LL_LRHGE-0.110416647*wave_glrlm_64_HL_LRLGE+0.006647641*wave_glo_64_HH_Kurtosis*

And we compared the Rad-scores from the training and validation cohort, respectively (Figure S2 A-B). The cutoff value was: 0.633.

Task 2

Rad-score calculation formula:

*Radscore=0.91727087+0.22847109*Length_to_width_ratio+0.41777488*text_Ng_8_vox_1_glszm_SZE-0.47085703*text_Ng_8_vox_1_glszm_GLN+0.18005714*text_Ng_8_vox_1_glszm_GLV-0.13915114*text_Ng_16_vox_1_glcm_Correlation-0.15659459*text_Ng_16_vox_1_glrlm_SRLGE-0.39416731*text_Ng_64_vox_1_glrlm_LRLGE+0.23764550*text_Ng_64_vox_1_glrlm_RLV-0.05174552*wave_glrlm_8_LL_SRLGE+0.01542789*wave_glrlm_8_HL_GLN-0.03812738*wave_glcm_8_LH_Correlation-0.11581065*wave_ngtdm_8_HH_Busyness-1.03630076*wave_glszm_16_LL_SZE+0.07135257*wave_glo_16_LH_Kurtosis-0.12035504*wave_ngtdm_32_LL_Complexity+0.06422489*wave_glszm_32_HL_LZHGE+0.06871394*wave_glrlm_64_LL_GLV+0.06814292*wave_glszm_64_LL_LZLGE+0.11571409*wave_glszm_64_LL_LZHGE-0.24486282*wave_glo_64_HL_Variance+0.09832092*wave_glszm_64_HL_LZLGE+0.39509210*wave_glcm_64_HH_Correlation*

And we compared the Rad-scores from the training and validation cohort respectively (Figure S2 C-D). The cutoff value was: 0.491.

3. Supplementary Figures and Tables

3.1 Supplementary Figures

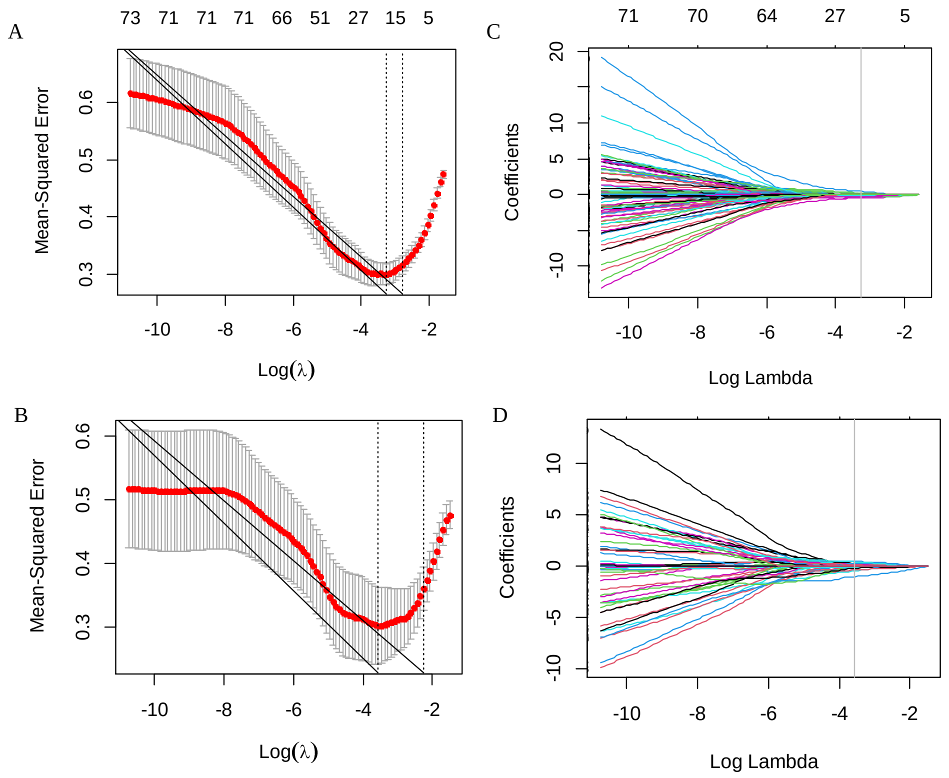

Figure S1: Feature selection using the least absolute shrinkage and selection operator (LASSO) binary logistic regression model for tow tasks. (A-B) Tuning parameter (Lambda) selection in the LASSO model used 10-fold cross-validation via minimum criteria for task 1 and 2, respectively. The gray line in the figure is the partial likelihood estimate corresponding to the optimal value of lambda. The optimal lambda value of 0.061 (task 2: 0.104) was chosen. (C-D) LASSO coefficient profiles of the features of task 1 and 2, respectively. A vertical line was plotted at the optimal lambda value, which resulted in 17 (task 1) and 22 (task 2) features with nonzero coefficients.

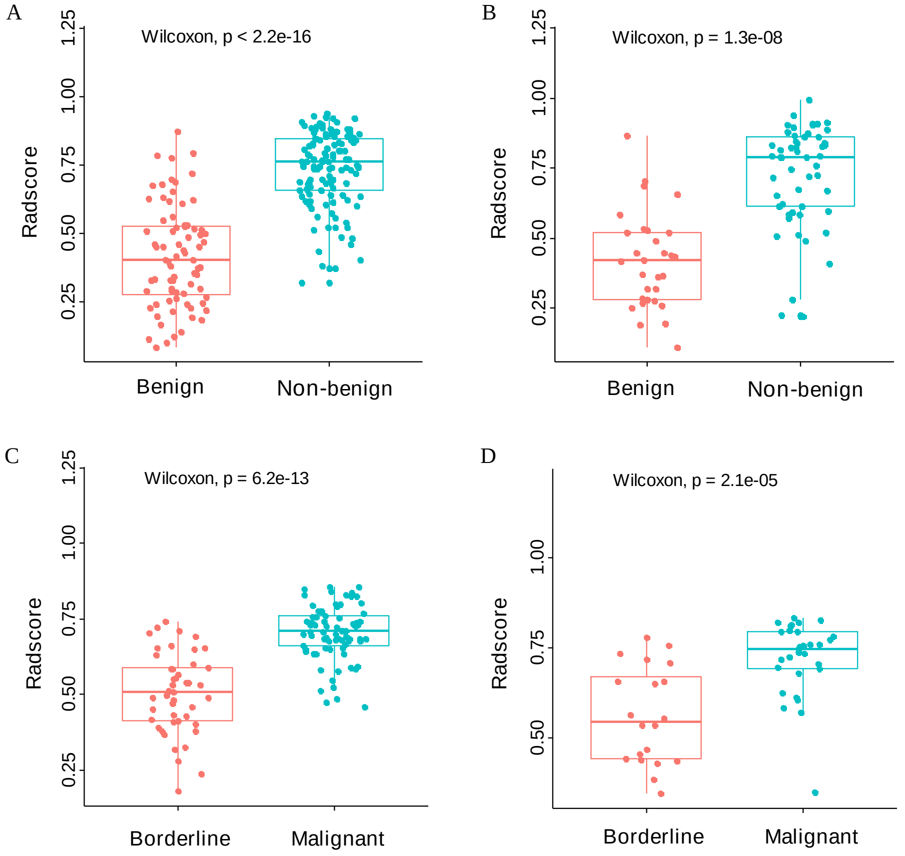

4.

5.

Figure S2: (A) The radscore from the training cohort for task 1. (B) The radscore from the validation cohort for task 1. (C) The radscore from the training cohort for task 2. (D) The radscore from the validation cohort for task 2.

3.2 Supplementary Tables

Table S1. Texture features used in this study.

Texture type	Reference(s)	Texture name
**GLCM**[a]	(Haralick et al 1973)	Energy
		Contrast
		Correlation
		Homogeneity
		Variance
		Sum Average
		Entropy
		Dissimilarity
		Auto Correlation
**GLRLM**[b]	(Galloway 1975)	Short Run Emphasis (SRE)
		Long Run Emphasis (LRE)
		Gray-Level Non-uniformity (GLN)
		Run-Length Non-uniformity (RLN)
		Run Percentage (RP)
	(Chu et al 1990)	Low Gray-Level Run Emphasis (LGRE)
		High Gray-Level Run Emphasis (HGRE)
	(Dasarathy and Holder 1991)	Short Run Low Gray-Level Emphasis (SRLGE)

		Short Run High Gray-Level Emphasis (SRHGE)
		Long Run Low Gray-Level Emphasis (LRLGE)
		Long Run High Gray-Level Emphasis (LRHGE)
	(Thibault et al 2009)	Gray-Level Variance (GLV)
		Run-Length Variance (RLV)
**GLSZM**[c]	(Galloway 1975, Thibault et al 2009)	Small Zone Emphasis (SZE)
		Large Zone Emphasis (LZE)
		Gray-Level Non-uniformity (GLN)
		Zone-Size Non-uniformity (ZSN)
		Zone Percentage (ZP)
	(Chu et al 1990, Thibault et al 2009)	Low Gray-Level Zone Emphasis (LGZE)
		High Gray-Level Zone Emphasis (HGZE)
	(Dasarathy and Holder 1991,	Small Zone Low Gray-Level Emphasis (SZLGE)
	Thibault et al 2009)	Small Zone High Gray-Level Emphasis (SZHGE)
		Large Zone Low Gray-Level Emphasis (LZLGE)
		Large Zone High Gray-Level Emphasis (LZHGE)
	(Thibault et al 2009)	Gray-Level Variance (GLV)

		Zone-Size Variance (ZSV)
**NGTDM**[d]	(Amadasun and King 1989)	Coarseness
		Contrast
		Busyness
		Complexity
		Strength

[a] GLCM: Gray-level co-occurrence matrix.
[b] GLRLM: Gray-level run-length matrix.
[c] GLSZM: Gray-level size zone matrix.
[d] NGTDM: Neighborhood gray-tone difference matrix.

Table S2. Performance comparison among radiomics, clinics and combination of radiomics and clinics in the training cohort of each task

		AUC (95% CI)	ACC	SEN	SPE
**Task 1**	Radiomics	0.907 (0.863-0.950)	0.852 (0.795-0.899)	0.822 (0.711-0.898)	0.870 (0.794-0.922)
	Clinics	0.817 (0.765-0.868)	0.760 (0.694-0.818)	0.656 (0.549-0.749)	0.854 (0.768-0.914)
	Combination	0.937 (0.905-0.969)	0.878 (0.823-0.920)	0.871 (0.765-0.936)	0.881 (0.808-0.930)
**Task 2**	Radiomics	0.891 (0.833-0.950)	0.836 (0.758-0.897)	0.766 (0.616-0.872)	0.880 (0.780-0.940)
	Clinics	0.815 (0.740-0.890)	0.730 (0.642-0.806)	0.594 (0.464-0.712)	0.879 (0.761-0.946)
	Combination	0.924 (0.876-0.971)	0.828 (0.749-0.890)	0.700 (0.566-0.808)	0.952 (0.856-0.987)

AUC area under the receiver operator characteristic curves, ACC accuracy, SEN sensitivity, SPEC specificity.

Table S3. Comparison of performance of the fixed training/validation split and the 10-fold cross-validation

		AUC (95% CI)	ACC (95% CI)	SEN (95% CI)	SPE (95% CI)
**Task 1**	Fixed training/validation split	0.877 (0.798-0.957)	0.843 (0.747-0.914)	0.758 (0.574-0.883)	0.900 (0.774-0.963)
	10-fold cross-validation	0.899	0.869	0.869	0.878
**Task 2**	Fixed training/validation split	0.839 (0.725-0.952)	0.824 (0.691-0.916)	0.923 (0.621-0.996)	0.790 (0.622-0.899)
	10-fold cross-validation	0.872	0.860	0.890	0.836

AUC area under the receiver operator characteristic curves, ACC accuracy, SEN sensitivity, SPE specificity.
