# Supplementary material for: Diagnosis of Ovarian Neoplasms Using Nomogram in Combination With Ultrasound Image-Based Radiomics Signature and Clinical Factors
Source: Front Genet. 2021 Sep 28;12:753948. doi: 10.3389/fgene.2021.753948 (PMC8505695; doi:10.3389/fgene.2021.753948)
Supplement: Supplementary file 2 [file DataSheet1.ZIP › Assignment.docx]

Age： 0: <50 years old 1: ≥50

CA125: 0: <500 IU/L 1: ≥500 IU/L

Tumor side： 1: Bilateral 0: Unilateral

Family history of cancer： 1: Yes 0: No

Ascites： 1: Yes 0: No
